# Association of circulating proprotein convertase subtilisin/kexin type 9 levels and the risk of incident type 2 diabetes in subjects with prediabetes: a population-based cohort study

**DOI:** 10.1186/s12933-020-01185-3

**Published:** 2020-12-10

**Authors:** Jie Shi, Weiwei Zhang, Yixin Niu, Ning Lin, Xiaoyong Li, Hongmei Zhang, Renming Hu, Guang Ning, Jiangao Fan, Li Qin, Qing Su, Zhen Yang

**Affiliations:** 1 Department of Endocrinology, Xinhua Hospital, Shanghai Jiao Tong University School of Medicine, Shanghai, China; 2 Institute of Endocrinology and Diabetology, Department of Endocrinology and Metabolism, Huashan Hospital, Fudan University, Shanghai, China; 3 Shanghai National Clinical Research Center for Endocrine and Metabolic Diseases, Key Laboratory for Endocrine and Metabolic Diseases of the National Health Commission of PR China, Shanghai Institute of Endocrine and Metabolic Diseases, Ruijin Hospital, Shanghai Jiao Tong University School of Medicine, Shanghai, China; 4 Department of Gastroenterology, Shanghai Key Laboratory of Children’s Digestion and Nutrition, Xinhua Hospital, Shanghai Jiao Tong University School of Medicine, Shanghai, China

**Keywords:** PCSK9, Type 2 diabetes, Prediabetes

## Abstract

**Background:**

Proprotein convertase subtilisin/kexin type 9 (PCSK9) regulates cholesterol metabolism by targeting the low-density lipoprotein receptor. Recent studies have shown that circulating PCSK9 is associated with glucose homeostasis and insulin resistance. The aim of this study was to examine the association of circulating PCSK9 levels and risk for the development of type 2 diabetes in individuals with prediabetes.

**Methods:**

A population-based prospective study was conducted among 4205 Chinese subjects with prediabetes (average age 56.1 ± 7.5 years). Incident type 2 diabetes was diagnosed according to 2010 American Diabetes Association criteria. Circulating PCSK9 levels were measured using a commercially available enzyme-linked immunosorbent assay (ELISA). The association of circulating PCSK9 levels with the risk of incident type 2 diabetes was assessed by Cox regression analysis.

**Results:**

During a median follow-up period of 3.1 years, 568 subjects developed type 2 diabetes. Baseline circulating PCSK9 levels were significantly higher in female subjects developing incident type 2 diabetes than in those not developing incident type 2 diabetes (p < 0.001). In female subjects, the risk of incident type 2 diabetes was significantly higher in the highest PCSK9 quartile group (hazard ratio 2.16; 95% confidence interval 1.16–4.04) than in the lowest quartile group after adjustments for age, body mass index, waist circumference, C-reactive protein, γ-glutamyltransferase, triglycerides, low-density lipoprotein cholesterol, systolic blood pressure, and homeostatic model assessment of insulin resistance score. No significant association was observed between PCSK9 and incident type 2 diabetes in male subjects.

**Conclusion:**

Elevated circulating PCSK9 levels are associated with an increased incidence of type 2 diabetes in female subjects with prediabetes.

## Background

Prediabetes, an intermediate metabolic state of hyperglycemia that involves higher than normal glucose that is lower than the threshold for clinical diabetes, is a heterogeneous subclinical status of diabetes. In prediabetic patients, risk progresses to diabetes up to 74% over the lifetime [1].

Proprotein convertase subtilisin/kexin type 9 (PCSK9) is the ninth member of the proprotein convertase family [2]. PCSK9 is a serine protease produced primarily by the liver and the intestine, but circulating PCSK9 is mainly derived from the liver according to the results of animal studies [3]. The finding that PCSK9 acts as a regulator pathway for hepatic low-density lipoprotein receptor (LDLR) degradation through an endosomal/lysosomal pathway sheds light on newly uncovered issues regarding LDL cholesterol (LDL-C) homeostasis [4]. Apart from this classical pathway, recent studies have described other roles played by PCSK9 in atherosclerosis via a variety of nonclassical mechanisms that involve inflammatory, apoptotic, and immune pathways [5].

The existence of a close relationship between lipid and glucose metabolism has supported research of the possible participation of PCSK9 in glucose homeostasis. However, evidence from genetic [6, 7], epidemiological [8, 9], and anti-PCSK9 clinical studies [10, 11] is rather inconclusive and controversial. For example, data from a genetic study showed that *PCSK9* variants were associated with an increased risk of type 2 diabetes [6]. Moreover, multiple epidemiological studies have found that circulating PCSK9 levels are higher in patients with diabetes than in those without diabetes [8], and significant positive associations between PCSK9 and fasting glucose, fasting insulin, and homeostasis model assessment of insulin resistance (HOMA-IR) have been reported [8, 12]. More recently, a cohort study found that high circulating PCSK9 is associated with an increased risk of diabetes in individuals after renal transplantation [13]. Evidence from cell and animal studies has also shown that downregulating PCSK9 ameliorates lipid and glucose metabolism in type 2 diabetes [14]. However, genetic deletion of *PCSK9* in mice is associated with impaired insulin secretion and glucose intolerance [15], and a small sample cohort study reported that plasma PCSK9 levels were not associated with the conversion to diabetes [9]. Indeed, there is currently no general agreement about the relationship between PCSK9 and diabetes, and further clarification is needed. Moreover, little is known about the link between circulating PCSK9 levels and incident type 2 diabetes in prediabetic individuals [9].

Therefore, the current study aimed to investigate the relationship between circulating PCSK9 levels and the risk of incident type 2 diabetes in a large-scale prospective cohort of Chinese adults with prediabetes.

## Methods

### Study cohort

**Fig. 1:**
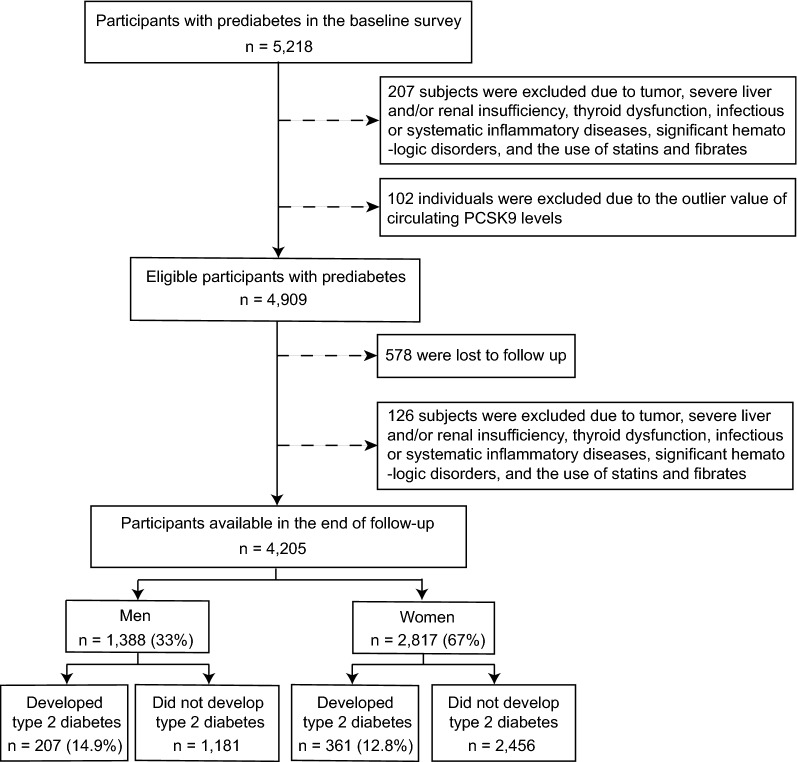
Overview of the study design

Participants were recruited from the China Cardiometabolic Disease and Cancer Cohort (4C) Study, a nationwide population-based prospective cohort study investigating associations of metabolic factors with specific clinical outcomes, including diabetes, cardiovascular disease, cancer, and all-cause mortality. The study design has been described in detail [16, 17]. The data presented in this study are based on subsamples from Chongming District in Shanghai, China. From May to November 2011, a total of 5218 prediabetic subjects aged 40 to 73 years were enrolled in the study. Prediabetes is defined as a fasting plasma glucose (FPG) level of 5.6–6.9 mmol/L (100–125 mg/dL), a postloading plasma glucose (PPG) level of 7.8–11.0 mmol/L (140–199 mg/dL), or a glycated hemoglobin (HbA_1c_) level of 5.7–6.4% (39–46 mmol/mol) according to the American Diabetes Association (ADA) guidelines from 2010. From June to November 2014, the subjects were invited for follow-up assessments. Of the 5218 subjects who initially participated at baseline, 4909 were enrolled in the follow-up study. Subjects with the following conditions were excluded from this study: tumor, severe liver and/or renal insufficiency, thyroid dysfunction, significant hematologic disorders, infectious or systematic inflammatory diseases, use of lipid-lowering agents (statins and fibrates), and PCSK9 outliers, which were defined as the extreme value (lower or upper 1% of the distribution) or repeated measurements coefficient of variation > 15%. The final study population consisted of 4,205 participants (mean ± SD age 56.1 ± 7.5 years), as depicted in Fig. 1.

The study protocol was approved by the Ethics Committee of Xinhua Hospital Affiliated to Shanghai Jiao Tong University School of Medicine (No. LLWYH-2011-32). Written informed consent was obtained from all participants.

### Data collection

At baseline, a questionnaire, physical examination, anthropometric measurements, and laboratory measurements were performed. Information about sociodemographic characteristics, medical histories, and lifestyle factors, including physical activity, smoking, and drinking habits, was collected by trained staff using a standard questionnaire. Physical activity was estimated using the short form of the International Physical Activity Questionnaire by adding questions regarding the frequency and duration of moderate and vigorous activities and walking [Guidelines for data processing and analysis of the International Physical Activity Questionnaire (IPAQ)]. Current smokers or drinkers were defined as individuals who had a regular smoking or drinking status in the past 6 months. All participants underwent anthropometrical measurements conducted by well-trained examiners using standard protocols. Body weight, standing height, and waist circumference were measured when participants were dressed in light clothes without shoes. BMI was calculated as the weight in kilograms divided by the square of height in meters (kg/m^2^). Blood pressure was consecutively measured three times within a 5-min interval using an automated electronic device (OMRON Model1 Plus; Omron Company, Kyoto, Japan), and the mean values were used for analysis. Waist circumference was measured at the umbilical level when the participant was in a standing position at the end of gentle expiration. According to the World Health Organization classifications, overweight was defined as a BMI ≥ 25 kg/m^2^; waist circumference reference values were 80 cm for women and 90 cm for men.

### Clinical and biochemical measurements

Prior to the baseline survey, the subjects were asked to fast overnight for at least 10 h, and fasting venous blood samples were collected by experienced nurses. A standard 75-g oral glucose tolerance test (OGTT) was carried out for all participants, and plasma glucose concentrations were determined at 0 and 2 h. The fasting and OGTT 2-h venous blood samples were collected into a routine tube and immediately processed by centrifugation at 4 °C for 10 min at 3000 relative centrifugal force. FPG, PPG, fasting serum insulin concentrations, triglycerides (TG), high-density lipoprotein cholesterol (HDL-C), LDL-C, total cholesterol (TC), alanine aminotransferase (ALT), aspartate aminotransferase (AST), and γ-glutamyltranspeptidase (GGT) were detected within 1 h of collection. Another anticoagulated venous blood sample (heparin) was collected for the measurement of HbA_1c_ within 4 h of collection.

Venous plasma glucose levels were assessed by the glucose oxidase method (ADVIA-1650 Chemistry System, Bayer, Leverkusen, Germany) and HbA_1c_ by high-performance liquid chromatography (BIO-RAD, D10, CA). Fasting insulin was determined by RIA (Linco Research, St. Charles, MO). Serum creatinine, TG, TC, HDL-C, LDL-C, ALT, AST, and GGT were measured with an autoanalyzer (Hitachi 7080; Tokyo, Japan). C-reactive protein (CRP) was determined by ELISA with a Duoset kit (R&D Systems, Minneapolis, MN). The abbreviated Modification of Diet in Renal Disease formula recalibrated for Chinese individuals was used to estimate the glomerular filtration rate, as expressed in milliliters per minute per 1.73 m^2^: estimated glomerular filtration rate (eGFR) = 186 * [serum creatinine * 0.011]^−1.154^ * [age]^−0.203^ * [0.742 if female] * 1.233, where serum creatinine is expressed as micromoles per liter and 1.233 is the adjusting coefficient for Chinese. The homeostasis model assessment-insulin resistance (HOMA-IR) was used to evaluate insulin resistance, and scores were calculated using the following equation: HOMA-IR = insulin (µU/mL) * glucose (mmol/L)/22.5.

### Measurement of circulating PCSK9 levels

Circulating levels of PCSK9 were measured using ELISA (DY3888; R&D Systems, Minneapolis, MN) according to the manufacturer’s instructions and compared with purified human PCSK9 standards. All samples were assayed in duplicate, and the mean intra- and interassay coefficients of variation were 2.24–8.75% and 4.12–10.83%, respectively.

### Prospective follow-up and end point definitions

In 2014, all subjects were asked to return to complete a 75-g OGTT and questionnaires for diagnosing new cases of diabetes. Questionnaires on current health status included questions about medication, hospital admission, and outpatient diabetes diagnosis. The endpoint was defined as incident type 2 diabetes based on the results of the OGTT and a review of all available hospital records. Incident type 2 diabetes included newly diagnosed and undiagnosed diabetes cases. Newly diagnosed diabetes was defined as a positive response from the participant to the question, “Have you been told that you have diabetes by a doctor since 2011?” New undiagnosed diabetes was defined according to the ADA 2010 criteria, as follows: FPG ≥ 7.0 mmol/L (≥ 126 mg/dL), 2-h PPG ≥ 11.1 mmol/L (≥ 200 mg/dL), or HbA_1c_ ≥ 6.5% (48 mmol/mol). We did not record the type of diabetes among incident cases, but given that all the participants in our study were at least 40 years old at enrollment, these participants were unlikely to have type 1 diabetes because fasting glucose was only mildly elevated and insulin levels were high normal (Table 1). All potential events of prediabetes and type 2 diabetes were independently adjudicated by two study physicians. In cases of disagreement, a consensus was achieved after a consultation with an endocrinologist.

**Table 1:** Baseline characteristics of subjects with or without incident type 2 diabetes

Variables	Total (n = 4205)	Incident type 2 diabetes		p value
		Without (n = 3637)	With (n = 568)	
Age (years)	56.1 ± 7.5	55.9 ± 7.4	57.1 ± 7.5	< 0.001
Male, n (%)	1388 (33.0)	1181 (32.5)	207 (36.4)	0.061
Current smoker, n (%)	555 (13.2)	468 (12.9)	87 (15.3)	0.109
Current drinker, n (%)	551 (13.1)	467 (12.8)	84 (14.8)	0.201
Physical activity, n (%)				0.367
Low	3020 (71.8)	204 (71.6)	416 (73.2)	
Middle	912 (21.7)	793 (21.8)	119 (21.0)	
High	273 (6.5)	240 (6.6)	33 (5.8)	
Educational attainment (years)				< 0.001
0–6	938 (22.3)	778 (21.4)	160 (28.2)	
7–9	2090 (49.7)	1819 (50.0)	271 (47.7)	
≥ 10	1177 (28.0)	1040 (28.6)	137 (24.1)	
Hypertension, n (%)	1106 (26.3)	917 (25.2)	189 (33.3)	< 0.001
IFG, n (%)	2120 (50.4)	1724 (47.4)	396 (69.7)	< 0.001
IGT, n (%)	1899 (45.2)	1509 (41.5)	390 (68.7)	< 0.001
iHbA_1c_, n (%)	2819 (67.0)	2365 (65.0)	454 (79.9)	< 0.001
SBP (mmHg)	128.78 ± 19.72	127.67 ± 20.00	135.88 ± 16.03	< 0.001
DBP (mmHg)	80.6 ± 10.6	80.27 ± 10.76	82.33 ± 9.49	< 0.001
BMI (kg/m^2^)	24.15 ± 4.62	24.04 ± 4.71	24.89 ± 3.87	< 0.001
WC (cm)	83.2 ± 8.5	82.7 ± 8.3	86.1 ± 8.7	< 0.001
Male	86.3 ± 8.5	86.0 ± 8.4	88.4 ± 8.6	0.004
Female	81.6 ± 8.4	81.1 ± 8.2	84.9 ± 8.7	< 0.001
FPG (mmol/L)	5.86 ± 0.49	5.82 ± 0.48	6.13 ± 0.46	< 0.001
PPG (mmol/L)	7.43 ± 1.67	7.29 ± 1.66	8.35 ± 1.69	< 0.001
HbA_1c_ (%)	5.68 ± 0.38	5.67 ± 0.38	5.76 ± 0.36	< 0.001
HbA_1c_ (mmol/mol)	38.57 ± 4.18	38.42 ± 4.20	39.52 ± 3.94	< 0.001
Insulin (mU/L)	6.7 (4.4–9.0)	6.7 (4.8–8.8)	7.7 (5.2–10.3)	< 0.001
HOMA-IR	1.78 (1.31–2.47)	1.74 (1.29–2.37)	2.21 (1.42–2.78)	< 0.001
CRP (mg/L)	1.46 (0.76–2.65)	1.33 (0.71–2.39)	1.61 (0.83–2.95)	< 0.001
HDL-C (mmol/L)	1.23 ± 0.32	1.23 ± 0.33	1.22 ± 0.31	0.498
LDL-C (mmol/L)	2.65 ± 0.75	2.64 ± 0.74	2.68 ± 0.82	0.238
Total cholesterol (mmol/L)	4.68 ± 1.00	4.66 ± 1.00	4.78 ± 1.06	0.008
TG (mmol/L)	1.35 (0.96–1.95)	1.34 (0.95–1.87)	1.48 (1.04–2.36)	< 0.001
ALT (U/L)	14 (10–20)	13 (10–19)	16 (12–22)	< 0.001
AST (U/L)	19 (16–24)	18 (12–24)	22 (17–28)	< 0.001
GGT (U/L)	20 (13–32)	19 (13–31)	26 (16–40)	< 0.001
Cr (mmol/L)	66.78 ± 17.61	66.77 ± 18.28	66.87 ± 12.55	0.899
eGFR (mL/min/1.73 m^2^)	111.28 ± 12.84	111.42 ± 12.91	110.42 ± 12.44	0.085
PCSK9 (ng/mL)	285.62 ± 98.52	283.68 ± 97.09	298.04 ± 103.87	0.001
Male	277.50 ± 97.57	278.44 ± 97.69	272.13 ± 97.52	0.391
Female	289.62 ± 98.80	286.20 ± 97.67	312.90 ± 103.63	< 0.001

IFG defined as FBG 100–125 mg/dL (5.6–6.9 mmol/L). IGT defined as PPG 140–199 mg/dL (7.8–11.0 mmol/L). iHbA1c defined as HbA1c > 5.7% (> 39 mmol/mol)

*ALT* alanine aminotransferase, *AST* aspartate transaminase, *BMI* body mass index, *CRP* C-reactive protein, *Cr* serum creatinine, *DBP* diastolic blood pressure, *eGFR* estimated glomerular filtration rate, *FPG* fasting plasma glucose, *GGT* γ-glutamyltransferase, *HbA*_*1c*_ glycated hemoglobin, *HDL-C* high-density lipoprotein cholesterol, *HOMA-IR* homeostasis model assessment-insulin resistance, *IFG* impaired fasting glucose, *IGT* impaired glucose tolerance, *iHbA*_*1c*_ impaired HbA_1c_, *LDL-C* low-density lipoprotein cholesterol, *PPG* postprandial plasma glucose, *SBP* systolic blood pressure, *TC* total cholesterol, *TG* triglycerides, *WC* waist circumference

### Statistical analysis

Continuous variables with a normal distribution are presented as means ± SDs; variables with a skewed distribution were log-transformed to approximate normality before analysis, and the results are shown as medians (interquartile range). Categorical variables are reported as frequencies (%). The subjects were divided into two groups according to incident type 2 diabetes status. For comparisons between groups, we performed a two-tailed independent-samples Student’s t test for normally distributed variables and a Mann–Whitney U test for variables with highly skewed distributions. The chi-squared test was applied to compare categorical variables. Correlation coefficients between baseline circulating PCSK9 levels and metabolic parameters were calculated using Spearman correlation analysis. Multivariate Cox regression was carried out to determine the potential association between PCSK9 levels and the risk of incident type 2 diabetes. We also used restricted cubic splines to flexibly model the association of circulating PCSK9 levels on a continuous scale and the risk of incident type 2 diabetes. Hazard ratios (HRs) and 95% confidence intervals (CIs) for the relationship between PCSK9 and the incidence of type 2 diabetes were generated with multivariate Cox regression models. Model 1 was adjusted for age; model 2 was adjusted for the variables in model 1 plus smoking, drinking, physical activity, and educational attainment; model 3 was adjusted for the variables in model 2 plus BMI; model 4 was adjusted for the variables in model 3 plus waist circumference; and model 5 was adjusted for the variables in model 4 plus TG, LDL-C, systolic blood pressure (SBP), ALT, AST, GGT, CRP, and HOMA-IR. PCSK9 categories depended on sex-specific quartiles using the lowest quartile group as the reference.

Several risk factors may affect the association between PCSK9 levels and incident type 2 diabetes, particularly waist circumference, CRP, LDL-C, and insulin resistance. Therefore, subgroup analysis was performed to assess whether the association between PCSK9 levels and the risk of incident type 2 diabetes was robust in the presence of potential confounders. Subgroups were divided according to age (< 65 years versus ≥ 65 years), BMI (< 25 kg/m^2^ versus ≥ 25 kg/m^2^), waist circumference (< 80 cm versus ≥ 80 cm for women and < 90 cm versus ≥ 90 cm for men), CRP (< 3.0 mg/L versus ≥ 3.0 mg/L), LDL-C [< 3.37 mmol/L (130 mg/dL) versus ≥ 3.37 mmol/L (130 mg/dL)], insulin resistance status (present versus absent), and menopausal status (premenopause versus postmenopause).

We conducted two sensitivity analyses. First, we repeated the analysis and restricted prediabetes for participants who met at least two of the three glycemic criteria for prediabetes established by the ADA in 2010. Second, we evaluated the effect of excluding incident cases ascertained only on the basis of a single glycemic abnormality.

P values of < 0.05 were considered statistically significant in all of the analyses. All analyses were performed using SPSS software version 25.0 (SPSS Inc., Chicago, IL) and R version 3.6.1.

## Results

### Baseline characteristics of subjects with or without incident type 2 diabetes

Table 1 summarizes the baseline characteristics of 4205 subjects who were stratified according to the presence or absence of incident type 2 diabetes after follow-up. The mean age of the subjects was 56.1 ± 7.5 years. Notably, 33.0% of the subjects were men, and 26.3% had hypertension. Individuals with incident type 2 diabetes were older and had higher BMI, waist circumference, TG, ALT, AST, GGT, and CRP levels and hypertension prevalence.

### Distribution of baseline circulating PCSK9 levels

**Fig. 2:**
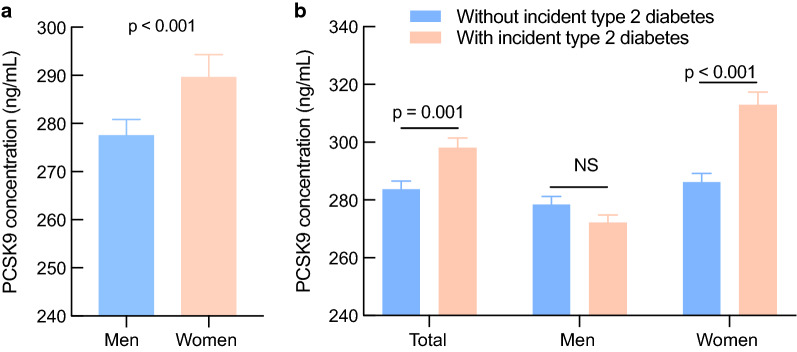
Baseline circulating PCSK9 levels in men and women (**a**) and in subjects with or without incident type 2 diabetes (**b**). Data are shown as the mean ± standard error of the mean (SEM). NS, no significance

Circulating PCSK9 levels were higher in females than in males (289.62 ± 98.80 ng/mL vs. 277.50 ± 97.57 ng/mL, p < 0.001) (Fig. 2a). Moreover, higher baseline PCSK9 levels were observed in female subjects with incident type 2 diabetes than in those without incident type 2 diabetes (312.90 ± 103.63 ng/mL vs. 286.20 ± 97.67 ng/mL, p < 0.001), but no significant differences were observed in males (Fig. 2b). In addition, circulating PCSK9 levels were significantly higher in postmenopausal women than in premenopausal women (299.54 ± 100.14 ng/mL vs. 261.66 ± 89.40 ng/mL, p < 0.001).

### Association of circulating PCSK9 levels with clinical characteristics

Table 2 represents the associations between circulating PCSK9 levels and clinical characteristics obtained from bivariate correlation analysis. In the univariate model, circulating PCSK9 levels correlated positively with age, waist circumference, FPG, HOMA-IR, SBP, LDL, TC, TG, ALT, AST, GGT, and CRP and inversely with eGFR in all subjects. Intriguingly, according to partial correlation analysis, a positive association of circulating PCSK9 with FPG, insulin, HOMA-IR, and CRP was observed only in female subjects after adjustment for age, smoking, drinking, physical activity, and educational attainment (Additional file 1: Table S1). Furthermore, there was a significantly positive correlation between PCSK9 and FPG, PPG, and HbA_1c_ at reexamination in females, representing a longitudinal association (Additional file 1: Table S2).

**Table 2:** Spearman correlation analysis between baseline circulating PCSK9 and clinical characteristics

Variables	Circulating PCSK9					
	Total		Men		Women	
	r	p value	r	p value	r	p value
Age	0.092*	0.001	0.077	0.109	0.197*	< 0.001
BMI	0.052	0.058	− 0.022	0.647	0.092*	0.006
Waist circumference	0.079*	0.004	− 0.018	0.707	0.144*	< 0.001
FPG	0.175*	< 0.001	0.116*	0.015	0.227*	< 0.001
PPG	− 0.011	0.679	− 0.105	0.107	0.037	0.275
HbA_1c_	− 0.051	0.067	− 0.102	0.060	− 0.035	0.293
Insulin	0.045	0.105	− 0.069	0.151	0.093*	0.006
HOMA-IR	0.078*	0.005	− 0.041	0.400	0.133*	< 0.001
CRP	0.108*	0.002	0.062	0.273	0.126*	< 0.001
SBP	0.100*	< 0.001	0.085	0.077	0.129*	< 0.001
DBP	0.023	0.404	0.037	0.441	0.039	0.248
HDL-C	− 0.083	0.056	− 0.072	0.075	0.092	0.059
LDL-C	0.163*	< 0.001	0.128*	0.008	0.176*	< 0.001
Total cholesterol	0.248*	< 0.001	0.254*	< 0.001	0.239*	< 0.001
Triglycerides	0.190*	< 0.001	0.188*	< 0.001	0.203*	< 0.001
ALT	0.176*	< 0.001	0.127*	0.008	0.225*	< 0.001
AST	0.185*	< 0.001	0.214*	< 0.001	0.191*	< 0.001
GGT	0.168*	< 0.001	0.229*	< 0.001	0.207*	< 0.001
Cr	0.028	0.313	0.102*	0.033	0.073*	0.031
eGFR	− 0.060*	0.031	− 0.096*	0.045	− 0.045	0.188

*BMI* body mass index, *FPG* fasting plasma glucose, *PPG* postprandial plasma glucose, *HbA*_*1c*_ glycated hemoglobin, *HOMA-IR* homeostasis model assessment-insulin resistance, *CRP* C-reactive protein, *SBP* systolic blood pressure, *DBP* diastolic blood pressure, *HDL-C* high-density lipoprotein cholesterol, *LDL-C* low-density lipoprotein cholesterol, *ALT* alanine transaminase, *AST* aspartate transaminase, *GGT* γ-glutamyltransferase, *Cr* serum creatinine, *eGFR* estimated glomerular filtration rate

^*^ p < 0.05

### Association between circulating PCSK9 levels and incident type 2 diabetes

**Fig. 3:**
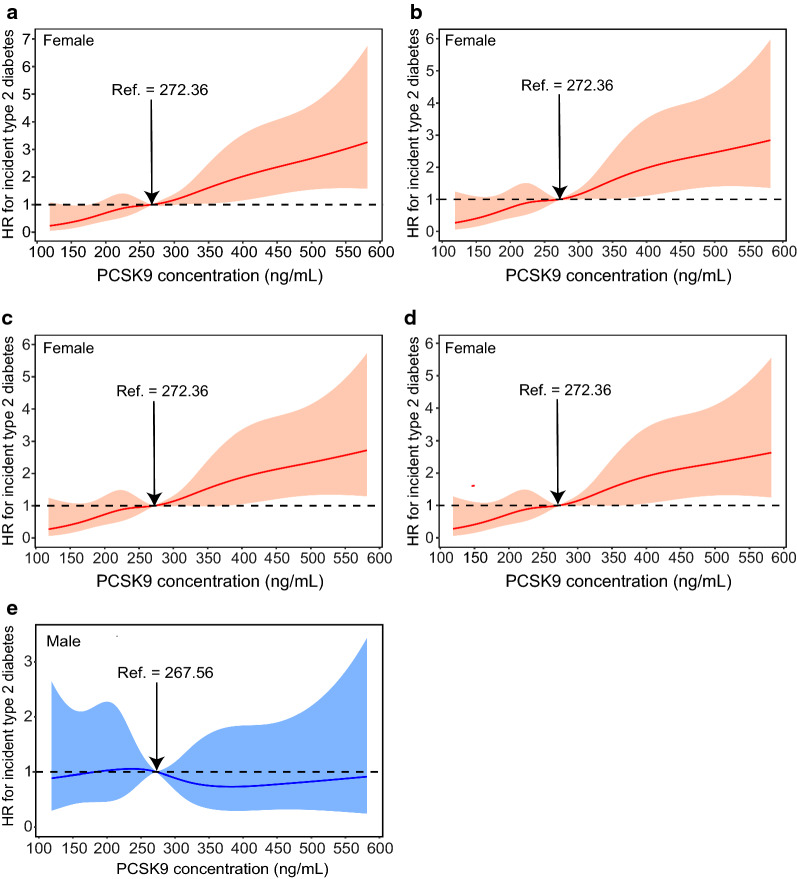
PCSK9 levels on a continuous scale and risk of incident type 2 diabetes in females (**a**–**d**) and males (**e**). Data were fit by a Cox regression model that was based on restricted cubic splines with five knots at the 5th, 35th, 50th, 65th, and 95th centiles. The reference point was a sex-specific median PCSK9 level of 272.36 ng/mL for females and 267.56 ng/mL for males. Hazard ratios are indicated by solid lines and 95% CIs by shaded areas. **a** Model 1 was adjusted for age, smoking, drinking, physical activity, and educational attainment. **b** Model 2 was adjusted for the variables in model 1 plus BMI. **c** Model 3 was adjusted for the variables in model 2 plus waist circumference. **d** Model 4 was adjusted for the variables in model 3 plus TG, SBP, ALT, AST, GGT, CRP, and HOMA-IR. **e** The crude model. For graphs (**a**) to (**d**), p value < 0.001 for the overall association and > 0.1 for nonlinearity for all outcomes. For graph (**e**), p value > 0.05 for the overall association and > 0.1 for nonlinearity

**Fig. 4:**
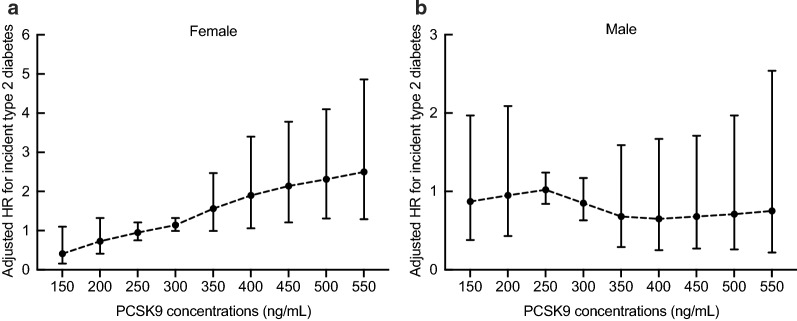
HRs (95% CI) for the 3.1-year risk of incident type 2 diabetes at different levels of PCSK9 (150–550 ng/mL) were analyzed by multivariable Cox regression models with restricted cubic splines after adjusting for age, smoking, drinking, physical activity, educational attainment, BMI, waist circumference, TG, LDL-C, SBP, ALT, AST, GGT, CRP, and HOMA-IR. Although there is overlap of confidence intervals in adjusted HRs at different PCSK9 concentrations, it appears that the trend is generally increasing with significant link for females. The reference standard was a sex-specific median PCSK9 level of 272.36 ng/mL for females and 267.56 ng/mL for males; p values < 0.001 for the overall association in females and > 0.05 in males

During a median follow-up period of 3.1 years, 568 (13.5%) subjects with prediabetes developed type 2 diabetes. Multivariate Cox regression models with restricted cubic spline analyses revealed that the risk of incident type 2 diabetes increased with increasing PCSK9 level on a continuous scale in female subjects (Fig. 3a–d) after adjusting for potential confounders. The p values were < 0.001 for the overall association and > 0.1 for nonlinearity for all outcomes. However, no significant association between circulating PCSK9 and the risk of incident type 2 diabetes was observed in males (Fig. 3e). Above the inflection point at 375 ng/mL, the risk of incident type 2 diabetes increased with increasing PCSK9 levels in female subjects (1.90 [1.06-3.40] at 400 ng/mL, 2.14 [1.21-3.78] at 450 ng/mL, 2.31 [1.31-4.10] at 500 ng/mL, and 2.50 [1.29-4.86] at 550 ng/mL) (Fig. 4).

### Subgroup analyses

We divided subjects into four groups based on sex-specific PCSK9 quartiles from this study to elucidate potential mechanisms. Compared with the low PCSK9 group, the high PCSK9 group were older and had high SBP, waist circumference, FPG, insulin, HOMA-IR, LDL-C, TC, TG, ALT, AST, GGT, and CRP, as well as a higher proportion of smokers and hypertension (Additional file 1: Table S3). We used Cox proportional hazards models with the lowest PCSK9 quartile group (PCSK9 < 220.20 ng/mL) as a reference to further assess the relationship between PCSK9 level and the risk of incident type 2 diabetes. As presented in Table 3, HRs for incident type 2 diabetes were higher with increasing PCSK9 quartile. In the highest PCSK9 quartile, the HR was 2.31 (95% CI 1.24–4.31; p < 0.001 for the trend) for incident type 2 diabetes after adjusting for age, lifestyle factors, physical activity, and educational attainment (model 2). Interestingly, further adjustment for BMI (model 3) and waist circumference (model 4) only slightly reduced the magnitude of the HRs for incident type 2 diabetes. Furthermore, significant differences remained (HR 2.16; 95% CI 1.16–4.04; p = 0.006) after additional adjustment for TG, LDL-C, SBP, ALT, AST, GGT, CRP, and HOMA-IR.

**Table 3:** Association of circulating PCSK9 with incident type 2 diabetes in females

	PCSK9				p for trend
	Q1 n = 704	Q2 n = 705	Q3 n = 704	Q4 n = 704	
Incidence of type 2 diabetes %	45 (6.4)	96 (13.6)	105 (14.9)	115 (16.4)	< 0.001
Model 1 HR (95% CI)	1.00	2.20 (1.17–4.14)	2.28 (1.21–4.26)	2.57 (1.39–4.77)	< 0.001
Model 2 HR (95% CI)	1.00	2.01 (1.06–3.79)	2.19 (1.17–4.10)	2.31 (1.24–4.31)	< 0.001
Model 3 HR (95% CI)	1.00	1.96 (1.04–3.71)	2.17 (1.16–4.07)	2.23 (1.20–4.16)	< 0.001
Model 4 HR (95% CI)	1.00	1.96 (1.04–3.70)	2.14 (1.14–4.01)	2.22 (1.19–4.13)	0.002
Model 5 HR (95% CI)	1.00	1.87 (0.99–3.53)	2.13 (1.14–4.00)	2.16 (1.16–4.04)	0.006

Model 1 was adjusted for age. Model 2 was adjusted for the variable in model 1 plus smoking, drinking, physical activity, and educational attainment. Model 3 was adjusted for the variables in model 2 plus BMI. Model 4 was adjusted for the variables in model 3 plus waist circumference. Model 5 was adjusted for the variables in model 4 plus TG, LDL-C, SBP, ALT, AST, GGT, CRP, and HOMA-IR. Subjects with a baseline circulating PCSK9 level in the lowest quartile group served as the reference group. Cutoff values in the four groups were Q1 < 220.20 ng/mL, Q2 220.21–272.36 ng/mL, Q3 272.37–342.61 ng/mL, and Q4 > 342.62 ng/mL for females

**Fig. 5:**
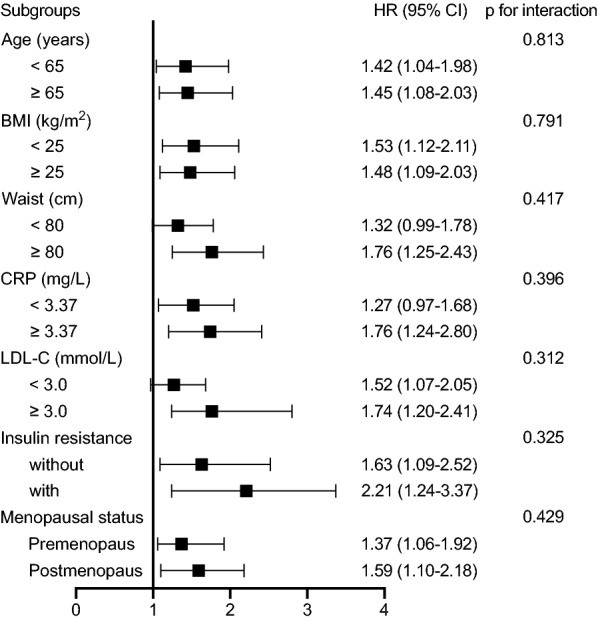
Stratified analyses of the associations between circulating PCSK9 and risk for incident type 2 diabetes in female individuals. Risk is estimated by 1-SD increased circulating PCSK9 levels. The hazard ratio (HR) and 95% confidence interval (CI) were obtained from multivariable Cox regression models after adjusting for age, smoking, drinking, physical activity, educational attainment, BMI, waist circumference, TG, LDL-C, SBP, ALT, AST, GGT, CRP, and HOMA-IR

In stratified analyses, the positive associations between PCSK9 and the risk of incident type 2 diabetes remained consistent across all subgroups (Fig. 5). The PCSK9-type 2 diabetes relationship was slightly stronger in individuals with higher waist circumference, higher levels of LDL-C, and insulin resistance than in their counterparts. However, no interaction was detected with any of the variables (all p for interaction > 0.10). Furthermore, we compared metabolic parameters according to the PCSK9 quartile group among the participants who developed diabetes. No significant difference in metabolic parameters was observed between the low and high PCSK9 groups, except for a higher level of TG in the high PCSK9 group (Additional file 1: Table S4).

### Sensitivity analyses

**Fig. 6:**
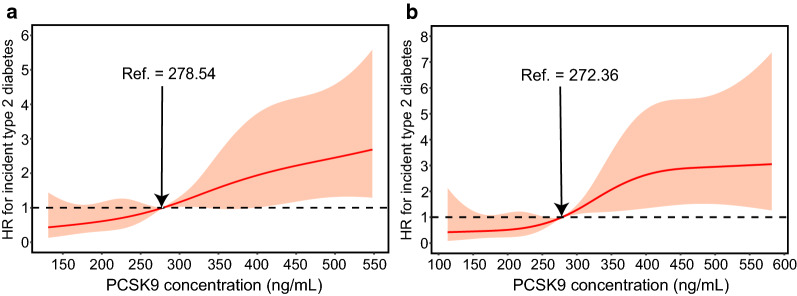
Sensitivity analysis of PCSK9 on a continuous scale and risk for incident type 2 diabetes in female subjects who met at least two of the three glycemic criteria for prediabetes (**a**) and the relationship between PCSK9 and confirmed type 2 diabetes in female subjects (**b**). Hazard ratios (HRs) are represented by solid lines and 95% CIs by shaded areas. The reference standard was a median PCSK9 level. The model was adjusted for age, smoking, drinking, physical activity, educational attainment, BMI, waist circumference, TGs, LDL-C, SBP, ALT, AST, GGT, CRP, and HOMA-IR; p values < 0.001 for the overall association and > 0.1 for nonlinearity for all outcomes

In total, 1,567 participants who met at least two of the three glycemic criteria for prediabetes were included in sensitivity analysis, including 277 (17.7%) participants who progressed to type 2 diabetes (Fig. 6a). We also performed another sensitivity analysis to assess the robustness of our findings only in confirmed cases (Fig. 6b). The results from the sensitivity analyses were consistent with the primary analyses.

## Discussion

In this population-based cohort study, we found a significantly positive association between circulating PCSK9 level and the risk of incident type 2 diabetes in female subjects with prediabetes. This association remained after extensively adjustment for potential confounders and stratification of several potential risk factors that may have an effect on the PCSK9-type 2 diabetes relationship. Conversely, no significant association was observed among male prediabetic subjects.

To the best of our knowledge, this is the largest and first study to undertake a longitudinal analysis of circulating PCSK9 and the development of type 2 diabetes in Asian individuals with prediabetes. Previous cross-sectional studies have shown higher PCSK9 levels in type 2 diabetes [13, 18]. PCSK9 is also increased in type 1 diabetes among younger subjects [19], and plasma PCSK9 levels increased significantly with glycemic control worsening [20]. Moreover, evidence from animal studies has shown that downregulating PCSK9 by polydatin ameliorates lipid and glucose metabolism [14]. Nonetheless, some investigations have failed to identify a relationship between PCSK9 and diabetes [9, 21]. Brouwers et al. [21] reported that glucose metabolism status per se is not associated with plasma PCSK9 levels, and longitudinal analyses by Ramin-Mangata et al. [9] showed that plasma PCSK9 levels were not significantly associated with new-onset diabetes risk. Such an ambiguous correlation may be explained in part by cross-sectional study design, limited sample size, different (non-Asian) populations, lack of stratification by sex, and especially incomplete adjustment for potential confounders such as the use of statins and fibrates, which are known to increase circulating PCSK9 levels.

We observed that PCSK9 concentrations were positively associated with glucose hemostasis, including FPG, insulin levels, and the HOMA-IR index in female subjects, in agreement with other data [8, 12]. Nevertheless, the exact molecular mechanisms by which PCSK9 facilitates the pathogenesis of diabetes are not yet clear. More intensive investigations are needed to clarify the precise mechanisms by which the PCSK9 pathway is involved in diabetes development.

The best-known function of PCSK9 is posttranslational regulation of LDLR on hepatocytes, which is the major route for LDL-C clearance from the circulation. In addition to from the canonical pathway, PCSK9 regulates lipid metabolism by intracellular endogenous PCSK9-induced LDLR degradation, VLDLR degradation, regulation of apolipoprotein B secretion, and lipoprotein(a) metabolism [5, 22]. PCSK9 is also associated with macrophage cholesterol efflux, apoptosis of endothelial cells, mitochondrial dysfunction, and inflammation [5], which contribute to metabolic disturbance. Furthermore, we found that PCSK9 was positively associated with ALT, AST, and GGT [23], liver function indices that are known as sensitive indicators of liver dysfunction and hepatic insulin resistance in diabetes [24, 25].

Previous studies have shown that PCSK9 acts as an independent predictor of the computed tomography angiography score, together with age, male sex, statins, interleukin-6, and leptin [26]; furthermore, serum PCSK9 levels have the potential to serve as a prescriptive biomarker for early arteriosclerosis in newly diagnosed type 2 diabetes [27]. In addition, the PCSK9 inhibitor alirocumab significantly reduces cholesterol and LDL levels regardless of sex among individuals with type 2 diabetes and atherosclerotic cardiovascular disease presenting high non-HDL-C/LDL-C levels [28], and alirocumab is an effective therapeutic option for patients with type 2 diabetes and mixed dyslipidemia [29]. Whether available PCSK9-targeting approaches may exert similar effects on glucose metabolism remains to be elucidated.

The most striking finding in the current study was the positive association of circulating PCSK9 levels with an increased risk of diabetes only in female subjects with prediabetes. Prior studies have also shown higher PCSK9 levels in females than in males, both in adults and children [8, 12, 19, 23]. Moreover, some studies found correlations between PCSK9 and a variety of clinical characteristics, including age, BMI, LDL-C, hepatic triglyceride content, glucose, blood pressure, and CRP, that were either stronger or only present in females [8, 18, 23]. These data suggest that the relationship between PCSK9 and the incidence of diabetes in individuals with prediabetes may be modified by sex. Nevertheless, the exact molecular mechanisms underlying such sex differences are not yet clear. The effect of sex on the association between PCSK9 and the development of diabetes needs to be delineated in more detail in future studies.

Knowler et al. [30] estimated that without intervention, 37% of individuals with prediabetes would develop diabetes within 4 years. The China Da Qing Diabetes Prevention Study 2 (CDQDPS) [31] reported a cumulative diabetes incidence of 66% among control group subjects who did not receive lifestyle intervention during the 6-year follow-up. During the 20-year follow-up, the cumulative diabetes incidence was 93% in the control group. Hence, differences in baseline PCSK9 levels in males with and without incident type 2 diabetes may be observed when the follow-up period is longer than 3.1 years. Along this line, further long-term studies are needed to determine the relationship between PCSK9 and the incidence of type 2 diabetes among male individuals.

Many studies in humans described PCSK9 plasma levels as being significantly higher in females than in males, but differences have also been observed in postmenopausal compared to premenopausal women and in pregnant compared to nonpregnant women, thus allowing us to hypothesize a role for sex hormones in PCSK9 synthesis and/or metabolism. Furthermore, animal and human studies have shown that PCSK9 is controlled by hormones such as insulin, glucagon, estrogen, growth hormone, and thyroid hormone [32]. To further examine the hormonal effect on PCSK9 concentrations, we compared circulating PCSK9 levels in pre- and postmenopausal women. In line with previous observations [8, 33], circulating PCSK9 levels were higher in postmenopausal women than in premenopausal women. It has been reported that circulating PCSK9 levels are reduced when endogenous estrogens are high in women [34], and pharmacologically increased estrogen levels have been shown to lower PCSK9 levels in animals and humans [35, 36]. Regardless, some studies have found that the difference in circulating PCSK9 levels between postmenopausal and premenopausal women appears to be independent of estrogen status [8], and estrogen at physiological concentrations does not affect human hepatocyte PCSK9 expression [33]. Overall, the underlying mechanism needs further elucidation.

It has been suggested that PCSK9 expression may be driven by insulin resistance, a common feature of metabolic syndrome [9]. A previous study found that dysbiosis of the gut microbiota exacerbates insulin resistance, possibly leading to a rise in PCSK9 expression [37]. Therefore, the relationship between insulin resistance and PCSK9 levels may be affected by gut microbiota dysbiosis. However, due to our study design, we did not analyze the difference in gut microbiota and its effect on the results. Nevertheless, our findings were maintained in analyses stratified by insulin resistance status in women. Further studies about the role of the gut microbiota in the PCSK9-diabetes relationship are needed.

The strengths of the current study include its large-scale, longitudinal follow-up of a high-risk population and extensive adjustment for key confounders and stratification of several potential risk factors that may have an effect on the PCSK9-type 2 diabetes relationship, including sex, waist circumference, LDL-C, HOMA-IR, and menopausal status. The results provide a reasonably accurate and unbiased estimate of the relationship between PCSK9 levels and incident type 2 diabetes.

### Limitations

Several potential limitations of the current study should be considered. First, the use of a single baseline PCSK9 measurement to predict outcomes was a simplified and practical approach, though it did not allow us to assess the relationship between changes in PCSK9 levels and the risk of incident type 2 diabetes. Second, due to the observational nature of the study, we were unable to determine causality based on our findings. Thus, further studies are necessary to clarify the mechanisms underlying the association of PCSK9 with type 2 diabetes and to determine whether high PCSK9 levels are a cause or a consequence of type 2 diabetes or both. Third, it is still unclear whether our findings in middle‐aged and older Chinese female individuals can be generalized to younger populations or individuals of other ethnicities. Further investigation should be undertaken among diverse populations. Fourth, we did not distinguish incident diabetes in terms of type, but the incidence of type 1 diabetes was very low, fasting glucose was only mildly elevated, and insulin levels were high-normal among the participants; accordingly, these participants are unlikely to develop type 1 diabetes. Fifth, our ascertainment of incident type 2 diabetes included individuals with undiagnosed diabetes. Although sensitivity analysis performed on confirmed cases was consistent with the original analysis, the PCSK9-diabetes relationship should also be exclusively analyzed using confirmed cases in future studies. Moreover, loss or gain of function of *PCSK9* gene mutations may have had an impact on the statistical results [38]. However, due to the design, we did not analyze *PCSK9* mutations in this study.

## Conclusion

In summary, our population-based longitudinal study identified a positive association between circulating PCSK9 levels and the risk of incident type 2 diabetes in female subjects with prediabetes.

## Supplementary Information

**Additional file 1: Table S1.** Partial correlation coefficients between baseline circulating PCSK9 and clinical characteristics. **Table S2.** Correlations between circulating PCSK9 at baseline and glucose parameters at reexamination. **Table S3.** Baseline characteristics of female participants according to PCSK9 quartiles. **Table S4.** Baseline characteristics of female participants according to PCSK9 quartiles in subjects with incident type 2 diabetes.

## Data Availability

The datasets used and/or analyzed during the current study are available from the corresponding author on reasonable request.

## Abbreviations

ALT: Alanine aminotransferase
AST: Aspartate transaminase
BMI: Body mass index
CRP: C-reactive protein
Cr: Serum creatinine
DBP: Diastolic blood pressure
eGFR: Estimated glomerular filtration rate
FPG: Fasting plasma glucose
GGT: γ-glutamyltransferase
HbA_1c_: Glycated hemoglobin
HDL-C: High-density lipoprotein cholesterol
HOMA-IR: Homeostasis model assessment-insulin resistance
IFG: Impaired fasting glucose
IGT: Impaired glucose tolerance
iHbA_1c_: Impaired HbA_1c_
LDL-C: Low-density lipoprotein cholesterol
PPG: Postprandial plasma glucose
SBP: Systolic blood pressure
TC: Total cholesterol
TG: Triglycerides
WC: Waist circumference
