# Supplementary material for: Association of circulating proprotein convertase subtilisin/kexin type 9 levels and the risk of incident type 2 diabetes in subjects with prediabetes: a population-based cohort study
Source: Cardiovasc Diabetol. 2020 Dec 10;19:209. doi: 10.1186/s12933-020-01185-3 (PMC7726879; doi:10.1186/s12933-020-01185-3)
Supplement: Supplementary file 1 — Additional file 1: Table S1. Partial correlation coefficients between baseline circulating PCSK9 and clinical characteristics. Table S2. Correlations between circulating PCSK9 at baseline and glucose parameters at reexamination. Table S3. Baseline characteristics of female participants according to PCSK9 quartiles. Table S4. Baseline characteristics of female participants according to PCSK9 quartiles in subjects with incident type 2 diabetes. [file 12933_2020_1185_MOESM1_ESM.docx]

**Additional data**

**Table S1.** Partial correlation coefficients between baseline circulating PCSK9 and clinical characteristics

| Variables | Circulating PCSK9 | | | | | |
| --- | --- | --- | --- | --- | --- | --- |
|  | Total | | Men | | Women | |
|  | r | p value | r | p value | r | p value |
| BMI | 0.050 | 0.091 | 0.028 | 0.583 | 0.064 | 0.077 |
| Waist circumference | 0.026 | 0.378 | -0.021 | 0.657 | 0.054 | 0.132 |
| FPG | 0.114* | <0.001 | 0.058 | 0.254 | 0.154* | <0.001 |
| PPG | -0.020 | 0.495 | -0.087 | 0.086 | 0.015 | 0.685 |
| HbA_1c_ | -0.044 | 0.130 | -0.082 | 0.105 | -0.039 | 0.278 |
| Insulin | 0.042 | 0.151 | -0.083 | 0.100 | 0.077* | 0.033 |
| HOMA-IR | 0.061* | 0.003 | -0.066 | 0.190 | 0.101* | 0.005 |
| CRP | 0.102* | 0.006 | 0.047 | 0.201 | 0.118* | 0.002 |
| SBP | 0.088* | <0.001 | 0.089 | 0.078 | 0.079* | 0.029 |
| DBP | 0.044 | 0.404 | 0.030 | 0.558 | 0.061 | 0.092 |
| HDL-C | -0.053 | 0.072 | -0.050 | 0.103 | -0.059 | 0.081 |
| LDL-C | 0.131* | <0.001 | 0.130* | 0.010 | 0.113* | 0.002 |
| Total cholesterol | 0.229* | <0.001 | 0.284* | <0.001 | 0.173* | <0.001 |
| Triglycerides | 0.138* | <0.001 | 0.180* | <0.001 | 0.107* | 0.003 |
| ALT | 0.105* | <0.001 | 0.035 | 0.488 | 0.159* | <0.001 |
| AST | 0.121* | <0.001 | 0.086 | 0.689 | 0.154* | <0.001 |
| GGT | 0.135* | <0.001 | 0.210* | <0.001 | 0.108* | 0.003 |
| Cr | 0.050 | 0.089 | 0.014 | 0.781 | 0.023 | 0.530 |
| eGFR | -0.007 | 0.824 | -0.015* | 0.038 | 0.043 | 0.234 |

* p <0.05. Partial correlation analysis model was adjusted for age, smoking, drinking, physical activity, and educational attainment. Abbreviations: BMI, body mass index; FPG, fasting plasma glucose; PPG, postprandial plasma glucose; HbA_1c_, glycated hemoglobin; HOMA-IR, homeostasis model assessment-insulin resistance; CRP, C-reactive protein; SBP, systolic blood pressure; DBP, diastolic blood pressure; HDL-C, high-density lipoprotein cholesterol; LDL-C, low-density lipoprotein cholesterol; ALT, alanine transaminase; AST, aspartate Transaminase; GGT, γ-Glutamyltransferase; Cr, serum creatinine; eGFR, estimated glomerular filtration rate.

**Table S2.** Correlations between circulating PCSK9 at baseline and glucose parameters at reexamination.

|  | Circulating PCSK9 | | | | | | | |
| --- | --- | --- | --- | --- | --- | --- | --- | --- |
| Variables | Spearman correlation coefficients | | | | Partial correlation coefficients | | | |
|  | Men | | Women | | Men | | Women | |
|  | r | p value | r | p value | r | p value | r | p value |
| FPG | 0.003 | 0.452 | 0.147 | <0.001 | 0.041 | 0.210 | 0.121 | <0.001 |
| PPG | 0.015 | 0.362 | 0.112 | <0.001 | 0.022 | 0.356 | 0.095 | <0.001 |
| HbA_1c_ | 0.012 | 0.401 | 0.114 | <0.001 | 0.033 | 0.310 | 0.073 | 0.003 |

Partial correlation analysis model was adjusted for age, smoking, drinking, physical activity, and educational attainment. Abbreviations: FPG, fasting plasma glucose; PPG, postprandial plasma glucose; HbA_1c_, glycated hemoglobin.

**Table S3.** Baseline characteristics of female participants according to PCSK9 quartiles

| Variables | Total  (n = 2,817) | PCSK9 | | | | p for trend |
| --- | --- | --- | --- | --- | --- | --- |
|  |  | Q1  (n = 704) | Q2  (n = 705) | Q3  (n = 704) | Q4  (n = 704) |  |
| Age (years) | 55.0±7.5 | 52.9±7.9 | 54.3±7.4 | 56.2±6.7 | 56.6±7.4 | <0.001 |
| Current smoker, n (%) | 156 (5.5) | 25 (3.6) | 32 (4.5) | 34 (4.8) | 65 (9.2) | 0.019 |
| Current drinker, n (%) | 298 (10.5) | 75 (10.7) | 73 (10.4) | 58 (8.2) | 92 (13.1) | 0.585 |
| Physical activity, n (%) | | | | | | 0.168 |
| Low | 2,048 (72.7) | 509 (72.3) | 515 (73.1) | 511 (72.6) | 513 (72.9) |  |
| Moderate | 602 (21.4) | 147 (20.9) | 151 (21.4) | 150 (21.3) | 154 (21.9) |  |
| High | 167 (5.9) | 48 (6.8) | 39 (5.5) | 43 (6.1) | 37 (5.2) |  |
| Educational attainment (years) | | | | | | <0.001 |
| 0-6 | 807 (28.6) | 159 (22.6) | 184 (26.1) | 230 (32.6) | 234 (33.2) |  |
| 7-9 | 1,394 (49.5) | 347 (49.3) | 354 (50.2) | 347 (49.3) | 346 (49.1) |  |
| ≥ 10 | 616 (21.9) | 198 (28.1) | 167 (23.7) | 127 (18.1) | 124 (17.6) |  |
| Hypertension, n (%) | 665 (23.6) | 134 (19.0) | 140 (19.9) | 188 (26.7) | 203 (28.8) | 0.005 |
| SBP (mmHg) | 126.42±20.53 | 121.60±23.98 | 127.20±17.81 | 128.55±20.55 | 128.33±18.56 | 0.001 |
| DBP (mmHg) | 79.18±10.90 | 78.91±10.14 | 79.02±11.60 | 78.86±11.62 | 79.92±10.18 | 0.712 |
| BMI (kg/m^2^) | 24.02±4.63 | 23.41±5.00 | 23.90±4.01 | 24.18±5.01 | 24.53±4.37 | 0.037 |
| WC (cm) | 81.4±8.5 | 79.1±8.1 | 81.5±8.6 | 81.6±8.5 | 83.3±8.4 | 0.013 |
| FPG (mmol/L) | 5.79±0.49 | 5.60±0.51 | 5.79±0.41 | 5.87±0.45 | 5.90±0.48 | <0.001 |
| PPG (mmol/L) | 7.42±1.69 | 7.37±1.65 | 7.24±1.79 | 7.55±1.72 | 7.51±1.58 | 0.218 |
| HbA_1c_ (%) | 5.70±0.39 | 5.73±0.33 | 5.67±0.50 | 5.72±0.33 | 5.69±0.36 | 0.395 |
| HbA1c (mmol/mol) | 38.82±4.22 | 39.15±3.61 | 38.49±5.41 | 38.97±3.63 | 38.69±3.98 | 0.395 |
| Insulin (mU/L) | 6.80 (5.0-9.1) | 6.70 (5.10-8.50) | 6.80 (4.90-9.00) | 7.30 (5.30-9.70) | 7.35 (5.30-9.60) | 0.044 |
| HOMA-IR | 1.83 (1.37-2.49) | 1.65 (1.28-2.26) | 1.78 (1.29-2.41) | 1.88 (1.44-2.61) | 1.92 (1.42-2.64) | 0.002 |
| CRP (mg/L) | 1.36 (0.74-2.52) | 1.29 (0.65-2.24) | 1.33 (0.73-2.46) | 1.39 (0.75-2.48) | 1.54 (0.80-2.75) | <0.001 |
| HDL-C (mmol/L) | 1.26±0.32 | 1.19±0.29 | 1.28±0.33 | 1.28±0.31 | 1.28±0.32 | 0.006 |
| LDL-C (mmol/L) | 2.68±0.77 | 2.45±0.71 | 2.63±0.75 | 2.81±0.80 | 2.82±0.76 | <0.001 |
| TC (mmol/L) | 4.71±1.02 | 4.30±0.89 | 4.66±1.03 | 4.91±1.00 | 4.96±1.03 | <0.001 |
| TG (mmol/L) | 1.31 (0.94-1.84) | 1.15 (0.82-1.57) | 1.19 (0.90-1.69) | 1.40 (1.01-1.88) | 1.43 (1.04-2.11) | <0.001 |
| ALT (U/L) | 13 (9-18) | 11 (7-15) | 12 (9-18) | 14 (10-19) | 15 (10-21) | <0.001 |
| AST (U/L) | 18 (15-23) | 16 (13-20) | 18 (14-23) | 19 (16-24) | 19 (17-24) | <0.001 |
| GGT (U/L) | 16 (12-24) | 14 (11-22) | 16 (12-24) | 19 (13-28) | 19 (13-29) | <0.001 |
| Cr (mmol/L) | 61.42±9.06 | 60.55±9.73 | 61.70±9.38 | 61.52±7.48 | 61.90±9.48 | 0.415 |
| eGFR (mL/min/1.73 m^2^) | 114.07±20.56 | 115.10±21.34 | 114.08±21.92 | 113.13±17.98 | 113.95±20.91 | 0.801 |
| PCSK9 (ng/mL) | 289.64±98.80 | 182.50±29.18 | 246.25±15.49 | 304.92±21.19 | 424.94±75.29 | <0.001 |

Abbreviations: ALT, alanine aminotransferase; AST, aspartate transaminase; BMI, body mass index; CRP, C-reactive protein; Cr, serum creatinine; DBP, diastolic blood pressure; eGFR, estimated glomerular filtration rate; FPG, fasting plasma glucose; GGT, γ-glutamyltransferase; HbA_1c_, glycated hemoglobin; HDL-C, high-density lipoprotein cholesterol; HOMA-IR, homeostasis model assessment-insulin resistance; LDL-C, low-density lipoprotein cholesterol; PPG, postprandial plasma glucose; SBP, systolic blood pressure; TC, total cholesterol; TG, triglycerides; WC, waist circumference.

**Table S4.** Baseline characteristics of female participants according to PCSK9 quartiles in subjects with incident type 2 diabetes

| Variables | Total  (n= 361) | PCSK9 | | | | p for trend |
| --- | --- | --- | --- | --- | --- | --- |
|  |  | Q1  (n= 45) | Q2  (n= 96) | Q3  (n= 105) | Q4  (n= 115) |  |
| Age (years) | 57.0±7.6 | 51.1±8.9 | 56.8±8.0 | 58.4±5.3 | 58.3±7.4 | 0.052 |
| Current smoker, n (%) | 23 (6.4) | 3 (6.7) | 6 (6.3) | 7 (6.7) | 7 (6.1) | 0.569 |
| Current drinker, n (%) | 34 (9.4) | 3 (6.7) | 13 (13.5) | 11 (10.5) | 7 (6.1) | 0.599 |
| Physical activity, n (%) | | | | | | 0.216 |
| Low | 265 (73.4) | 33 (73.3) | 70 (72.9) | 78 (74.3) | 84 (73.0) |  |
| Moderate | 80 (22.2) | 10 (21.4) | 21 (21.9) | 23 (22.0) | 26 (22.6) |  |
| High | 16 (4.4) | 2 (4.4) | 5 (5.2) | 4 (3.8) | 5 (4.3) |  |
| Educational attainment (years) | | | | | | 0.066 |
| 0-6 | 117 (32.4) | 6 (13.3) | 28 (29.2) | 41 (39.0) | 42 (36.5) |  |
| 7-9 | 170 (47.1) | 19 (42.2) | 46 (49.0) | 51 (48.6) | 54 (47.0) |  |
| ≥ 10 | 74 (20.5) | 20 (44.4) | 21 (21.9) | 13 (12.4) | 20 (17.4) |  |
| Hypertension, n (%) | 120 (33.2) | 10 (22.2) | 26 (27.1) | 40 (38.1) | 44 (38.3) | 0.153 |
| SBP (mmHg) | 134.21±16.55 | 125.55±12.76 | 137.89±18.03 | 134.88±15.26 | 133.93±16.91 | 0.140 |
| DBP (mmHg) | 81.22±9.57 | 80.45±9.63 | 80.31±10.46 | 82.45±7.86 | 81.17±10.31 | 0.832 |
| BMI (kg/m^2^) | 25.00±3.71 | 24.07±2.58 | 24.51±3.23 | 24.92±4.25 | 25.86±3.95 | 0.286 |
| WC (cm) | 85.0±8.5 | 79.6±8.4 | 85.4±8.6 | 83.88±8.5 | 87.8±8.4 | 0.089 |
| FPG (mmol/L) | 6.08±0.45 | 6.11±0.46 | 5.99±0.42 | 6.14±0.41 | 6.09±0.51 | 0.571 |
| PPG (mmol/L) | 8.30±1.72 | 8.20±1.96 | 8.32±1.62 | 8.23±1.76 | 8.39±1.75 | 0.977 |
| HbA_1c_ (%) | 5.79±0.37 | 5.87±0.35 | 5.70±0.39 | 5.85±0.35 | 5.77±0.36 | 0.357 |
| HbA1c (mmol/mol) | 39.74±4.02 | 40.67±3.77 | 38.83±4.29 | 40.38±3.86 | 39.56±3.98 | 0.357 |
| Insulin (mU/L) | 7.90 (5.40-10.45) | 7.05 (5.55-9.75) | 6.50 (5.00-9.23) | 7.90 (5.40-11.60) | 9.60 (5.75-11.10) | 0.129 |
| HOMA-IR | 2.24 (1.47-2.84) | 2.19(1.53-2.69) | 1.63(1.27-2.56) | 2.27(1.46-3.25) | 2.55(1.75-2.92) | 0.069 |
| CRP (mg/L) | 1.64 (0.81-2.90) | 1.49 (0.73-2.45) | 1.89 (0.76-3.14) | 1.72 (0.87-2.83) | 1.56 (0.77-3.16) | 0.114 |
| HDL-C (mmol/L) | 1.25±0.29 | 1.18±0.30 | 1.26±0.27 | 1.31±0.32 | 1.22±0.29 | 0.496 |
| LDL-C (mmol/L) | 2.75±0.88 | 2.59±1.02 | 2.56±0.60 | 2.83±0.95 | 2.90±0.94 | 0.183 |
| TC (mmol/L) | 4.86±1.14 | 4.37±1.33 | 4.65±0.92 | 5.03±1.08 | 5.06±1.23 | 0.132 |
| TG (mmol/L) | 1.47 (0.99-2.21) | 0.98 (0.71-1.69) | 1.36 (0.93-1.92) | 1.47 (1.01-2.35) | 1.54 (1.20-2.74) | 0.041 |
| ALT (U/L) | 16 (12-21) | 10 (8-18) | 16 (11-21) | 17 (14-21) | 16 (13-21) | 0.069 |
| AST (U/L) | 21 (17-26) | 16 (13-21) | 21 (16-29) | 24 (18-27) | 22 (18-25) | 0.056 |
| GGT (U/L) | 21 (15-33) | 16 (11-22) | 20 (14-34) | 24 (18-35) | 23 (14-35) | 0.088 |
| Cr (mmol/L) | 62.19±9.82 | 59.22±8.19 | 60.86±9.60 | 62.69±6.43 | 64.02±12.55 | 0.367 |
| eGFR (mL/min/1.73 m^2^) | 111.36±21.20 | 118.82±21.82 | 115.26±23.29 | 107.13±13.97 | 109.05±23.60 | 0.216 |
| PCSK9 (ng/mL) | 312.90±103.63 | 183.64±35.04 | 242.44±15.35 | 301.50±21.22 | 432.71±80.53 | <0.001 |

Abbreviations: ALT, alanine aminotransferase; AST, aspartate transaminase; BMI, body mass index; CRP, C-reactive protein; Cr, serum creatinine; DBP, diastolic blood pressure; eGFR, estimated glomerular filtration rate; FPG, fasting plasma glucose; GGT, γ-glutamyltransferase; HbA_1c_, glycated hemoglobin; HDL-C, high-density lipoprotein cholesterol; HOMA-IR, homeostasis model assessment-insulin resistance; LDL-C, low-density lipoprotein cholesterol; PPG, postprandial plasma glucose; SBP, systolic blood pressure; TC, total cholesterol; TG, triglycerides; WC, waist circumference.
